# SARNAclust: Semi-automatic detection of RNA protein binding motifs from immunoprecipitation data

**DOI:** 10.1371/journal.pcbi.1006078

**Published:** 2018-03-29

**Authors:** Ivan Dotu, Scott I. Adamson, Benjamin Coleman, Cyril Fournier, Emma Ricart-Altimiras, Eduardo Eyras, Jeffrey H. Chuang

**Affiliations:** 1 The Jackson Laboratory for Genomic Medicine, Farmington, CT, United States of America; 2 Research Programme on Biomedical Informatics (GRIB), Hospital del Mar Medical Research Institute (IMIM)–Pompeu Fabra University (UPF), Barcelona, Spain; 3 UCONN Health, Department of Genetics and Genome Sciences, Farmington, CT, United States of America; 4 Catalan Institution for Research and Advanced Studies (ICREA), Barcelona, Spain; University of Texas at Austin, UNITED STATES

## Abstract

RNA-protein binding is critical to gene regulation, controlling fundamental processes including splicing, translation, localization and stability, and aberrant RNA-protein interactions are known to play a role in a wide variety of diseases. However, molecular understanding of RNA-protein interactions remains limited; in particular, identification of RNA motifs that bind proteins has long been challenging, especially when such motifs depend on both sequence and structure. Moreover, although RNA binding proteins (RBPs) often contain more than one binding domain, algorithms capable of identifying more than one binding motif simultaneously have not been developed. In this paper we present a novel pipeline to determine binding peaks in crosslinking immunoprecipitation (CLIP) data, to discover multiple possible RNA sequence/structure motifs among them, and to experimentally validate such motifs. At the core is a new semi-automatic algorithm SARNAclust, the first unsupervised method to identify and deconvolve multiple sequence/structure motifs simultaneously. SARNAclust computes similarity between sequence/structure objects using a graph kernel, providing the ability to isolate the impact of specific features through the bulge graph formalism. Application of SARNAclust to synthetic data shows its capability of clustering 5 motifs at once with a V-measure value of over 0.95, while GraphClust achieves only a V-measure of 0.083 and RNAcontext cannot detect any of the motifs. When applied to existing eCLIP sets, SARNAclust finds known motifs for SLBP and HNRNPC and novel motifs for several other RBPs such as AGGF1, AKAP8L and ILF3. We demonstrate an experimental validation protocol, a targeted Bind-n-Seq-like high-throughput sequencing approach that relies on RNA inverse folding for oligo pool design, that can validate the components within the SLBP motif. Finally, we use this protocol to experimentally interrogate the SARNAclust motif predictions for protein ILF3. Our results support a newly identified partially double-stranded UUUUUGAGA motif similar to that known for the splicing factor HNRNPC.

## Author summary

RNA-protein binding is critical to gene regulation, and aberrant RNA-protein interactions play a role in a wide variety of diseases. However, molecular understanding of these interactions remains limited because of the difficulty of ascertaining the motifs that bind each protein. To address this challenge, we have developed a novel algorithm, SARNAclust, to computationally identify combined structure/sequence motifs from immunoprecipitation data. SARNAclust can deconvolve multiple motifs simultaneously and determine the importance of specific features through a graph kernel and bulge graph formalism. We have verified SARNAclust to be effective on synthetic motif data and also tested it on ENCODE eCLIP datasets, identifying known motifs and novel predictions. We have experimentally validated SARNAclust for two proteins, SLBP and ILF3, using RNA Bind-n-Seq measurements. Applying SARNAclust to ENCODE data provides new evidence for previously unknown regulatory interactions, notably splicing co-regulation by ILF3 and the splicing factor hnRNPC.

## Introduction

RNA-protein binding is a fundamental biological interaction vital to the diverse functions of RNA, including key roles in RNA splicing, translation, localization and stability [1–4]. However, the sequence features that determine affinity to RNA-binding proteins (RBPs) are unknown for most RBPs, including the vast majority of the hundreds of RBPs in the human proteome. Moreover, even for RBPs with known binding motifs, existing sequence motifs are only weakly predictive of which RNA regions will be bound. Deciphering these RNA binding features is crucial for mechanistic understanding of RNA-protein binding and understanding how RNA regulation impacts human health. RNA-protein interactions are known to play a role in a wide variety of diseases including muscular dystrophy, fragile X syndrome, mental retardation, Prader-Willi syndrome, retinitis pigmentosa, spinal muscular atrophy, and cancer [1–5].

Short single motifs are usually used to describe RNA-protein binding elements, e.g. as compiled in the RBPDB experimental database [6], but such motifs have often had poor predictive power. As an example, Hogan et al identified transcripts bound to 40 yeast RBPs and then searched their UTR regions for overrepresented sequences [7]. They were able to find statistically significant motifs for only 21 RBPs, and in many cases previously known motifs could not be found. This issue of poor predictive power for single motifs has continued even with finer resolution assays such as CLIP-seq, which can localize binding sites to within a few nucleotides [8,9]. For example, in CLIP-seq for *LIN28*–RNA interaction sites in human somatic and embryonic stem cells [10], the most overrepresented sequence motif (GGAGA) was found in less than 13% of the sites.

A possible explanation for this problem is that proteins have the potential to interact with multiple sequence motifs. For instance, it is known that Gemin5, a peripheral protein of the survival of motor neuron (SMN) complex in metazoan organisms [11–13], is responsible for recognition of the Sm site of snRNA [14,15]. This recognition is mediated by a WD40 repeat domain located in the N-terminus [16–18], yet there is also a bi-partite non-canonical RNA-binding domain at the C-terminus which modulates IRES-dependent translation [19,20]. However, computational methods to distinguish multiple motifs simultaneously have not been developed.

Existing computational approaches for RNA motif detection, which have been geared toward single motif discrimination, have had moderate success. RNA motif analysis has often been carried out by repurposing DNA motif finder tools such as MEME [21], PhyloGibbs [22] or cERMIT [23], but these methods cannot take into account RNA secondary structure. Many known RBPs do bind to single stranded RNA (ssRNA), but it remains unclear how much secondary structure impacts binding. Some methods have incorporated aspects of RNA structure, e.g. by biasing for single stranded regions [24,25] or searching over a limited set of structural contexts (paired, loop, unstructured, miscellaneous) [26–28]. However, the predictive power of these methods remains low, likely because of the limited number of considered contexts compared to the diversity of possible RNA structures. For example, Kazan et al. tested their algorithm on 9 RBP-interaction sets and found an average AUC value of only 0.64 [28]. Approaches that consider structural contexts using machine learning algorithms such as Support Vector Machines [29], Hidden Markov Models [30,31] or Deep Learning [32–34] have been developed. Some have improved cross-validation AUC values to 0.8 to 0.9 [34–36], though common caveats to current approaches are that they rely on immunoprecipitation training sets with uncertain specificity, that they have not been developed to handle multiple motifs, or that they have abstracted structural constraints rather than considering exact RNA structures.

Recently, Maticzka and colleagues developed the graph kernel-based GraphProt to handle sequence and structure together and applied it to learn motifs from CLIP-seq data [37], finding motifs that were predictive of binding for the protein PTB. However, this approach has not been tested for RBPs that bind to double stranded RNAs, and it is unknown whether the effectiveness would depend on the types of structures to which individual proteins bind. Moreover, GraphProt reports at most one motif and classifies the remaining data as noise. A more general approach would be to use clustering to allow for multiple possible motifs. A related method is GraphClust [38], which uses a sequence/structure graph kernel to cluster RNAs, and a recent extension called RNAscClust [39] incorporates orthologous sequence conservation to improve the RNA folding estimates into the clustering process. However, these methods are tailored to cluster non-coding RNAs, and it is unknown if they would be effective for the clustering of CLIP-seq sites.

Here, we propose a method, SARNAclust (Semi-Automatic RNA clustering), to cluster, as opposed to classify, RNA motifs that bind to a given RBP from CLIP-seq data. To our knowledge, this is the first approach to attempt to cluster CLIP-seq peaks in order to discover potentially multiple RNA motifs that bind to a given RBP. The most related approach we know of is AptaTrace [40], which uses clustering to identify multiple possible RNA motifs from HT-SELEX experiments. However, AptaTrace is not optimized for CLIP-seq data since it relies on k-mer context information during evolution of a sequence pool over multiple SELEX rounds, while CLIP-seq provides a static snapshot. Another recent method, RNAcompete-S [41] clusters multiple components that contribute to a single binding motif, but is not designed to handle distinct motifs arising from separate binding domains.

Here we present a novel pipeline to address these problems in RNA-protein motif identification. We first describe our pipeline, which consists of 3 steps (peak discovery, motif discovery and motif experimental validation), with particular attention to the novel computational motif discovery algorithm SARNAclust. Next we benchmark SARNAclust on synthetic data and validate our experimental protocol on a known double-stranded RNA motif. We then show SARNAclust motif predictions for a set of RBPs with eCLIP data and experimentally validate the motif of one such RBP. Finally, we discuss the results and implications of this pipeline in the Discussion section.

## Results

### Overview of the pipeline

We present a mixed computational/experimental pipeline to derive RNA motifs that bind to a given RBP based on immunoprecipitation data. The motivation for our pipeline is two-fold: first, to discover motifs where both sequence and structure are necessary; and second, to enable identification of more than one motif per RBP through optimized clustering over the CLIP peaks.

**Fig 1:**
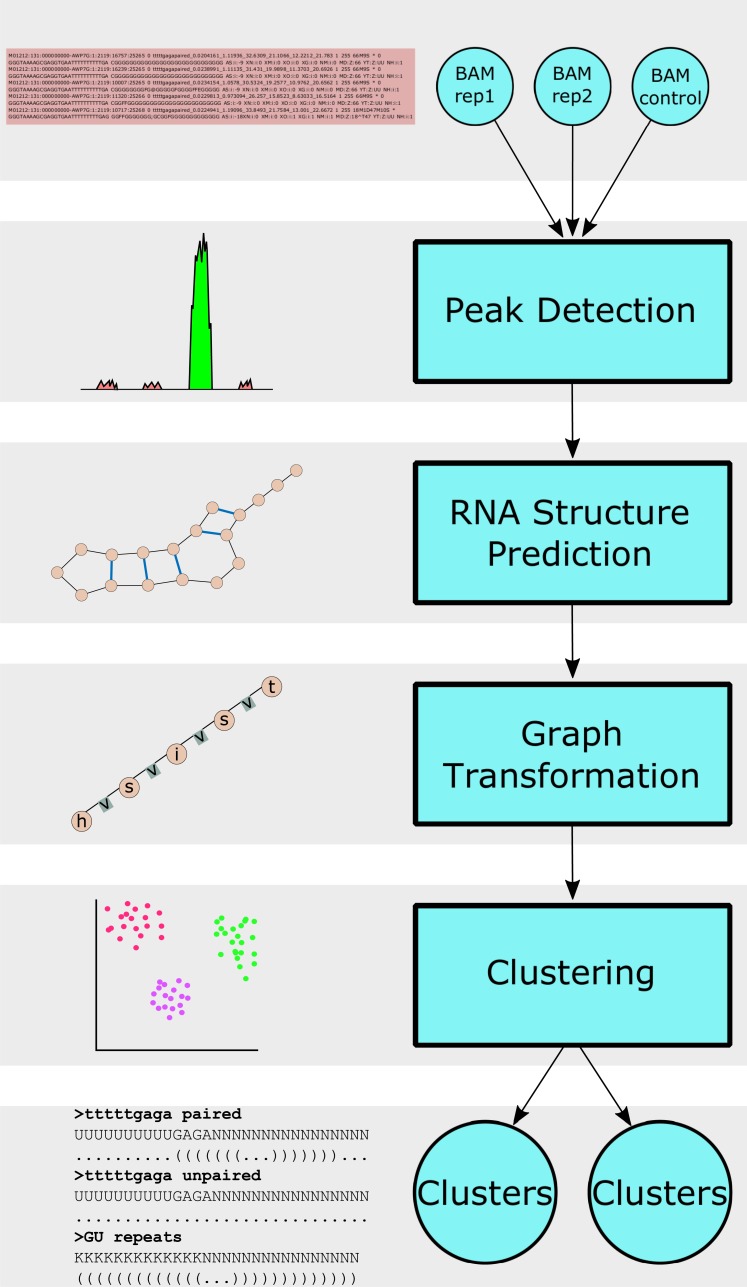
CLIPseq motif finding pipeline. Bam files for sample and control are processed through our peak detection module. The structure of each peak is calculated using RNAfold and the resulting sequence/structure peaks are clustered using SARNAclust.

Our complete software pipeline includes source code to process data files from a CLIP experiment (see Methods), to calculate secondary structure of the peaks using RNAfold, to cluster peaks according to sequence only, and to cluster peaks according to sequence/structure using SARNAclust. In addition, we provide a protocol for experimental validation of candidate motifs, including *in silico* design of instances of the motif using RNAiFold [42,43]. Fig 1 shows the flowchart of our pipeline. S1 Fig shows the flowchart of the peak analysis.

A key element of SARNAclust is the graph transformation that allows for the calculation of a similarity value between pairs of sequence/structures. These similarity values provide the input for the clustering of CLIP peaks. Flexible parameters in SARNAclust allow it to be used as a guidance system to identify well-supported motifs and test their key features.

#### SARNAclust

**Fig 2:**
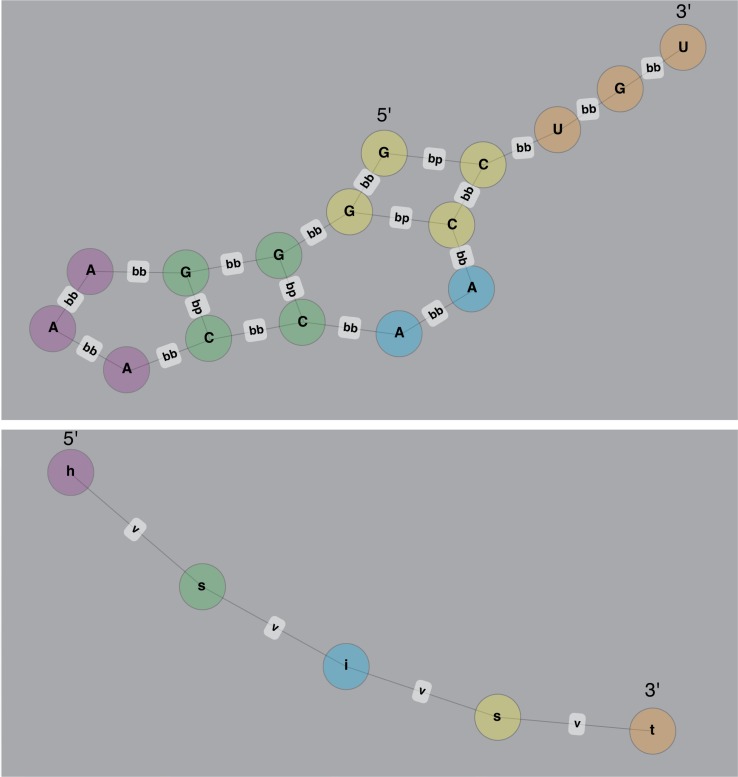
Graph formalisms. Complete graph and bulge graph sequence/structure representations used in SARNAclust. (top) complete graph and (bottom) bulge graph for example sequence/structure: GGGGAAACCAACCUGU and ((((. . .))..)). . . ..

Given a set of RNA sequence/structures calculated using RNAfold (or any other RNA structure prediction method), SARNAclust then clusters them. Similarities between pairs of sequence/structures are computed using the graph kernel in EdEN [44], which is equivalent to that used in GraphClust [38] and GraphProt [37]. However, parameter choices before applying this kernel for clustering are critical for accurate detection of motifs, which we have optimized as described below. To use the graph kernel we first transform the sequence/structures into graphs. Our pipeline allows for several different transformations based on either the complete graph or the bulge graph [45] (See Fig 2). The complete graph represents the secondary structure with all node connections between consecutive nucleotides or base pairs. The bulge graph is a condensed representation similar to the concept of abstract RNA shape [46].

In the complete graph (top) nodes are nucleotides and edges between nodes correspond to either base pairing (bp) or backbone links (bb). In the bulge graph (bottom) the structure is collapsed into structural elements, where “h” is hairpin loop, “i” is internal loop or bulge and “s” is stem (double stranded). “t” stands for the 3’ single stranded region.

SARNAclust provides the following options for graph transformations (see S2 Fig):

- Option 1: GraphProt-like consists of the complete graph plus a hypergraph, which is a less condensed version of the bulge graph.
- Option 2: GraphProt-like where the hypergraph is substituted by the bulge graph.
- Option 3: Bulge graph.
- Option 4: Bulge graph plus corresponding sequence in hairpin loops.
- Option 5: Bulge graph plus corresponding sequence in internal loops and bulges.
- Option 6: Bulge graph plus corresponding sequence in external loops.
- Option 7: Bulge graph plus corresponding sequence in base paired regions.
- Option 8: Bulge graph plus corresponding sequence in hairpin loops, internal loops and bulges.
- Option 9: Bulge graph plus corresponding sequence in all unpaired regions.
- Option 10: Bulge graph plus sequence everywhere.
- Option 11: Bulge graph plus complete graph where sequence in base paired regions is not taken into account.

We have provided a range of options because different RNA-binding proteins will vary in their dependence on different structural features, and in many cases such features may be known from the domains in the protein. These include options that exhaustively consider structure but may be more sensitive to noise (e.g. option 1) and those that reduce the set of considered structural contexts (e.g. option 11). For example, for options 9 or 11 to be suitable, the key binding element in the RNA should be expected to occur in unpaired regions but within a precise structural context. Overall, Options 1, 2 and 10 allow arbitrary sequence and structure motifs, but in decreasing order of resolution. The appropriate choice among these is an empirical question related to the noisiness of the data, which we investigate below. All other options consider sequence only within a specified structural context: i.e. internal loops, bulges, external loops, or double stranded regions. Among these, our prior expectation is that Options 9 and 11 would be the most effective, since they focus on sequence effects in single stranded regions. In the following section we show the effect of these transformations when applying our methods to synthetic data for different types of motifs.

Once the graph transformation has been applied, SARNAclust allows the user to apply one of several clustering algorithms and returns the clusters along with a consensus sequence/structure for each. The inputs to the clustering module are: 1) the file with the sequence/structures, 2) EdEN dimension [38], 3) EdEN radius, 4) the clustering method, and 5) the graph transformation option. To retrieve a motif from a cluster, we align the peaks of each cluster by both sequence and structure [47] and discard those where the alignment score is 0. Detailed descriptions of the pipeline, manuals, and source code are available at https://github.com/idotu/SARNAclust.

### Benchmarking on synthetic motif data

To test the effectiveness of SARNAclust, we generated 100 sequences for each of the 5 synthetic motifs in Table 1. We then combined these 500 sequences with 1000 random sequences to act as noise and then tested the ability of SARNAclust to sort these into separate clusters. Each synthetic motif corresponds to a hypothetical RNA motif that would bind a protein binding domain. The 5 motifs represent: a special structure with no sequence conservation (*special_structure*) or a conserved sequence within a certain structural context in a hairpin loop (*GAGA_in_Hairpin*), in a bulge (*AUG_in_Bulge*), in an external loop (*pyrimidine_tract*) or in a double stranded region (*GGUCG_in_left_stem*). Sequences for each motif were generated using RNAdualPF [48], which samples from the low energy ensemble of sequences compatible with the given structure and with the corresponding sequence constraints (see Table 1). The 1000 random sequences were generated uniformly randomly (i.e. sampling each nucleotide with 0.25 probability) with lengths distributed the same as the lengths of the synthetic motifs. All motif and random sequences can be found in S1 Data.

**Table 1:** Synthetic motifs used to test SARNAclust.

Motifs		
**special_structure**	NNNNNNNNNNNNNNNNNNNNNNNNNN	**Sequence**
	((.((((.. ((. . .))..))..))))	**Structure**
**AUG_in_Bulge**	NNNNAUGNNNNNNNNNNNNN	**Sequence**
	((((. . . (((. . .)))))))	**Structure**
**pyrimidine_tract**	NNNNNNNNNNNCCUCU	**Sequence**
	((((. . .)))). . . ..	**Structure**
**GAGA_in_Hairpin**	NNNNNGAGANNNNN	**Sequence**
	(((((. . . .)))))	**Structure**
**GGUCG_in_left_stem**	NNGGUCGNNNNNNNNNN	**Sequence**
	(((((((. . .)))))))	**Structure**

**Fig 3:**
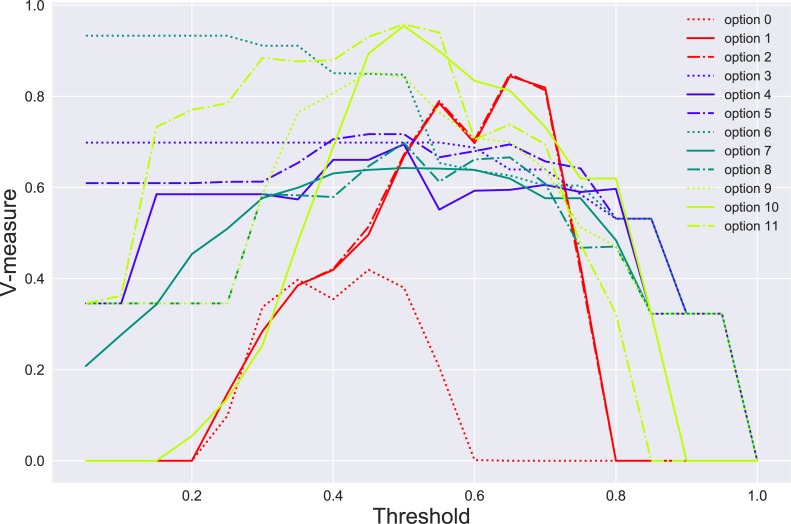
SARNAclust benchmarking. V-measure scores at different threshold values for each of the graph transformation options.

Clustering of these 1500 sequences indicated that SARNAclust was able to distinguish multiple clusters corresponding to the original motif groups. As a clustering method we used DBSCAN from the sklearn package, surveying over possible values for the threshold parameter that specifies the minimal similarity for two data points to be in the same cluster, and with graph kernel options R = 2 and D = 2. This threshold is a dissimilarity threshold—at a threshold of x, 2 sequence/structures cannot be in the same cluster if their similarity measure is less than 1-x. Fig 3 shows the different V-measure (a measure of clustering quality, see Methods) values for each graph transformation and each threshold value. S2 Data shows other measures of quality of clustering (see Methods) assessed by comparing the true cluster label for each sequence versus the one yielded by the clustering algorithm.

We observed that SARNAclust was able to recover each category of motif, though optimization of the choice of graph transformation enhanced detection of each motif class. As can be seen, use of each graph transformation yielded the corresponding motif at low to mid threshold values but false positives increased as the threshold parameter increased. For instance, option 4 finds the GAGA_in_hairpin motif easily at low thresholds. Although option 6 performs well, it benefits from the fact that most motifs do not have external loops and may not be as general as other options. For most motif instances option 6 is only able to use the bulge graph features to discriminate motif instances from one another. The GraphProt-like options (1 and 2) perform well at high threshold values, but cannot successfully cluster at low thresholds. This is due to the excess number of features specified in this graph transformation, making it difficult to cluster instances unless they are nearly identical. Option 2 contains fewer features than option 1 and thus performs better. Options 10 and 11 are simplified GraphProt-like versions and achieve the best results with v-measure values of over 0.95. Option 9 achieves high quality values as well, and for a large range of threshold, especially with respect to FMS.

For comparison, we also applied GraphClust to the same set of synthetic motifs. We note that we did not choose the GraphClust extension RNAscClust here because the folded structures are pre-determined for these synthetic designed sequences, and therefore the folding improvements of RNAscClust do not offer any advantages. We used the default GraphClust parameters except graph kernel R and D, which were set to the values we used in SARNAclust, and the minimum length of sequence was set so that all the 1500 sequences would be considered. GraphClust returns by default 5 clusters, which we would expect to correspond to the 5 synthetic motifs. GraphClust returns the seed and extended sequences for each instance, and we calculated several clustering quality measures for each (Adjusted Rand Index (ARI), Adjusted Mutual Information (AMI), Homogeneity Score (HS), Completeness Score (CS), V-measure score (VMS) and Fowlkes-Mallows score (FMS)).

SARNAclust outperformed GraphClust as shown in Table 2. Note that the GraphClust v-measure values in both scenarios are under 0.1, while SARNAclust with option 11 achieves better results for almost all thresholds, including >0.95 at threshold 0.5. This indicates that the difference between clustering CLIP peaks and RNAs are substantial enough that the SARNAclust provides superior performance over GraphClust. This is likely due to the large combinatorial complexity of GraphClust’s parameter space.

**Table 2:** GraphClust and SARNAclust results on synthetic data.

Type of Cluster	ARI	AMI	HS	CS	FMS	VMS
**GraphClust Seed**	0.017	0.011	0.012	0.365	0.685	0.024
**GraphClust Extended**	0.059	0.043	0.045	0.537	0.693	0.083
**SARNAclust Option 2**	0.64	0.56	0.56	0.82	0.84	0.84
**SARNAclust Option 11**	0.96	0.94	0.96	0.94	0.95	0.95

We also compared whether a classification-based approach to motif detection could identify the synthetic motifs as well as SARNAclust. To handle multiple motifs, we used classification to identify the best motif iteratively, at each step removing the sequences containing the prior best motif. For this comparison we chose RNAcontext [28], which uses classification to identify one motif at a time based on sequence and structure. Remarkably, the first iteration of RNAcontext could not find any of the synthetic motifs (S3 Fig). In fact, the sequence motif (of length 11) returned has a very low information content of 5 bits (when a completely fixed sequence of length 11 would have an information content of 22 bits). This is likely because the RNAcontext approach is not well-suited to the benchmark set as it contains multiple signals from distinct overrepresented motifs as well as noise. Interference among the motifs apparently causes RNAcontext to be unable to report any single motif with high confidence.

### Experimental validation with RNA Bind-N-Seq

As part of our pipeline for identifying binding motifs, we developed a targeted RNA Bind-N-Seq (RBNS) protocol [49] to experimentally test motif predictions. We first tested this protocol on Stem Loop Binding Domain Protein (SLBP), which binds a known motif [50] found in the 3’UTR of histone mRNAs. In the RNA Bind-N-Seq protocol, randomly generated 40-mers are tested for their efficiency in binding a protein. Because any RNA molecule of length 40 can form over 200 trillion secondary structures, it is not possible to fully sample this space. Therefore, we performed RBNS measurements of SLBP-RNA binding with several thousand designed sequences to ascertain the validity of our experimental validation approach.

To do this, we first used RNAiFold [43] (See Methods) to design four different types of sequences as illustrated in S1 Table (153, 4106, 4107 and 4106 sequences of each respectively). These sequences were chosen to test whether binding requires sequence conservation in the loop region or the stem region of the motif, and also whether relocation of the loop sequence to a different structural context (i.e. a bulge) could still lead to protein-RNA binding. We then performed RBNS in duplicate using purified GST-SBP-SLBP (Glutathione-S-transferase Streptavidin-Binding Peptide SLBP) to pull down the designed RNA sequences [49]. As a nonspecific binding control, we also performed RBNS with the same RNA against purified GST-SBP. Each protein was expressed in *E*. *coli* and affinity purified (S4 Fig), and pulled down RNA was reverse transcribed with a primer containing a 10 nt random sequence to enable collapsing of PCR duplicates during data analysis. The resulting cDNA was then PCR amplified to attach Illumina sequencing primers and indices.

**Fig 4:**
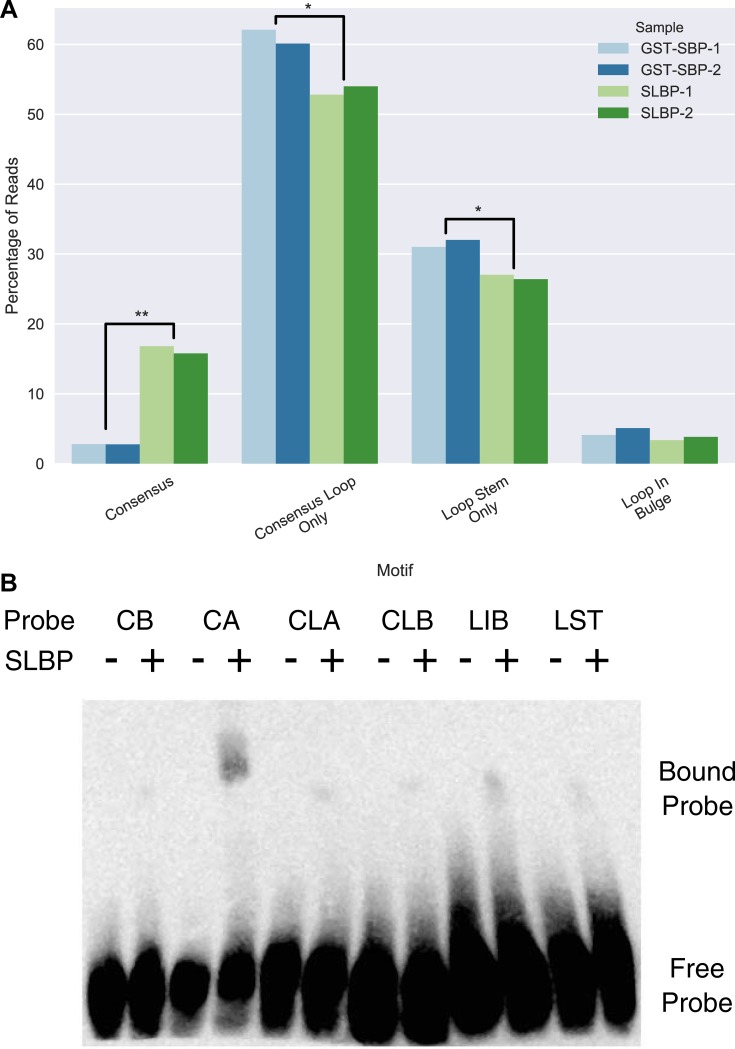
RBNS-like validation using known SLBP motif. a) Percentage shift in the sequences of each group of RNAs for SLBP RNA-bind-n-seq. GST-SBP samples are used as a non-specific binding control b) Gel shift results for select probes tested in the RBNS when incubated with purified GST-SBP-SLBP. The Consensus A (CA) probe shows more binding relative to Consensus B (CB), Consensus Loop Only A (CLA), Consensus Loop Only B (CLB), Loop In Bulge (LIB) and Loop Stem Only (LST). Sequences for each probe and their RBNS results can be found in S4 Data. * indicates p < 0.05, ** indicates p < 0.005 assessed by t-test.

Only the consensus motif [50] exhibited a clear shift from the control, indicating that the motif definition is specific and that all the variant versions of the motif have decreased binding. Fig 4A shows the difference between GST-SBP RBNS and GST-SBP-SLBP RBNS, quantified by the shift in percentage of reads of each type. Only the consensus motif has a significant enrichment with respect to the control (t-test p-val = 0.00147). To assess p-values of individual sequences, we used DEseq [51] to compare the read counts (S3 Data) of sequences in the pool to the controls. This analysis showed that only sequences from the consensus motif bind to SLBP significantly. Moreover, all but 7 of these consensus sequences are significantly overrepresented in the SLBP bound pool (Adjusted p-val > 0.01). Furthermore, S5 Fig shows the sequence logos for all the consensus sequences that bind or do not bind significantly, respectively. The logos indicate that long stretches of U’s near the apical region of the hairpin loop compromise binding affinity, which is to be expected since they are energetically unfavorable and therefore prone to render the hairpin unstable.

To further validate these results and the validity of our RBNS-like experimental protocol, we performed several gel shift experiments (Fig 4B). We incubated 6 RNA probes selected from the RBNS data with purified GST-SBP-SLBP. S3 Data shows the 6 selected sequences highlighted in red. These include 2 from the consensus binding group, one with strong binding affinity in the RBNS assay (consensus A) and one with no significant binding affinity (consensus B). There are also 4 extra sequences from the remaining types where the RBNS binding signal was not significant. As expected, only the consensus A sequence shows binding to SLBP, confirming our conclusions from the p-value analysis and validating the RBNS protocol.

### SARNAclust predicts novel motifs in ENCODE eCLIP data

Given these validations of the computational and experimental pipeline, we then applied SARNAclust to predict motifs from real immunoprecipitation data. First, we verified that SARNAclust could find the motif for SLBP. In order to do so, we downloaded SLBP eCLIP [52] data from the ENCODE project (www.encodeproject.org). After applying our peak discovery pipeline we were left with only 49 peaks, most of them (i.e. 35) found indeed in histone genes. After calculating the secondary structure of each peak using RNAfold, we ran SARNAclust.

**Fig 5:**
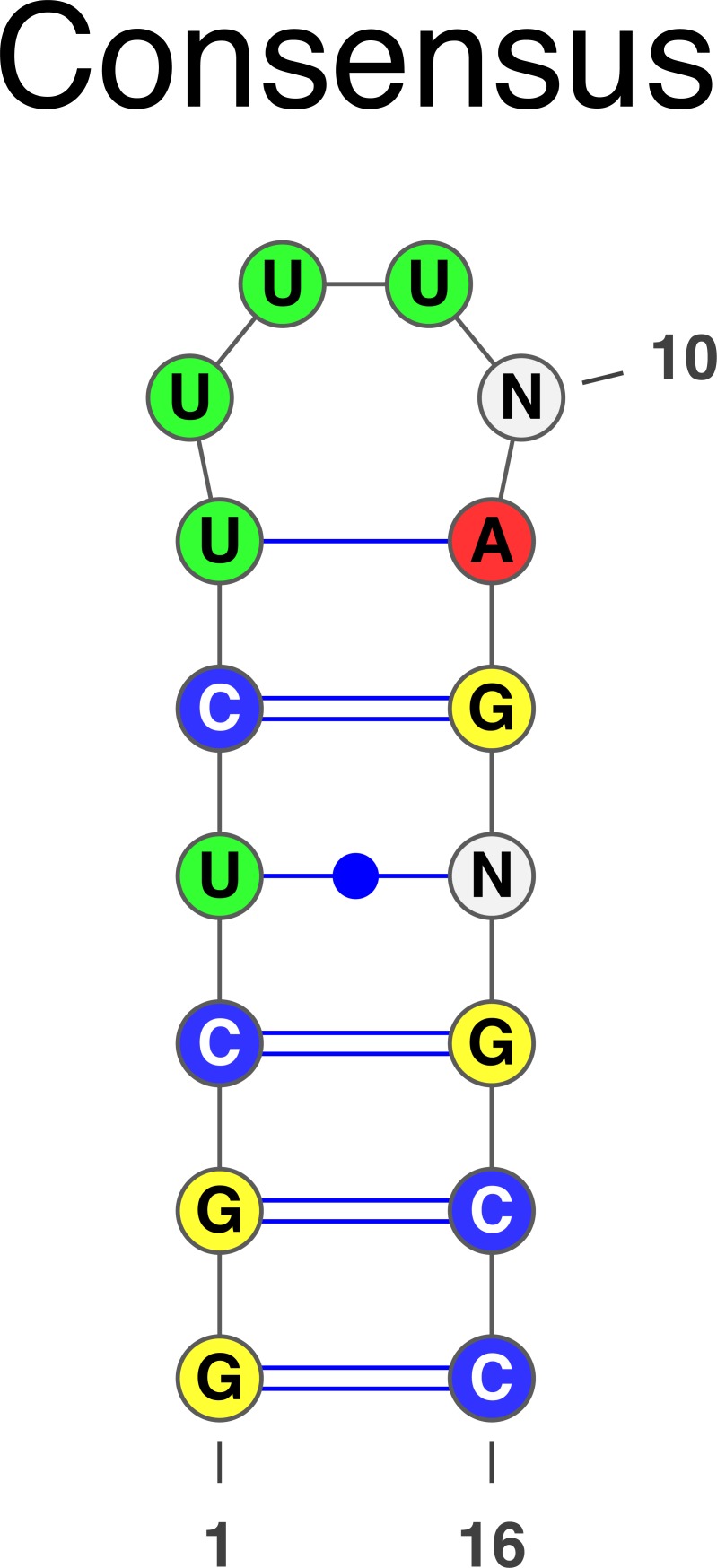
SARNAclust motif discovered for SLBP. Consensus sequence/structure motif found for SLBP by SARNAclust with graph transformation options 7 and 10.

Fig 5 shows the motif found for options 1, 2 and 10 at threshold 0.6, as well as option 7 at threshold 0.5. These are the most suited options since they account for sequence in double stranded regions, which is important for SLBP. Options 7 and 10 yield less specific clustering, meaning that they need higher thresholds (i.e. the clusters contain more sequences) to find the motif, consistent with their being coarser representations of the sequence/structure. As can be seen, the motif found is very similar to the canonical motif [50]. No other clusters were found, showing SARNAclust is effective even if only one motif exists.

We then used SARNAclust to predict motifs for several RBPs from the ENCODE project, which is generating RNA crosslinking immunoprecipitation assays that are expected to eventually cover >200 known human RNA Binding Proteins using eCLIP [52]. We downloaded a set of 20 RBPs from ENCODE eCLIP experiments at www.encodeproject.org, each with 2 replicates and a control. We selected these RBPs due to the fact that they contain either double stranded RNA binding domains or unknown RNA binding domains. We discarded all helicases since they are known to promiscuously bind to double-stranded RNAs with no clear motif. We identified novel motifs with SARNAclust using the same graph kernel parameters as in the synthetic data section (R = 2,D = 2) and the same DBSCAN algorithm for clustering. We used options 9,10, 11 for their overall performance on the synthetic data, along with option 2 for its similarity to GraphClust, at the best performing thresholds (0.3–0.55).

Table 3 shows the list of RBPs chosen for this study, along with their RNA binding domains and the number of peaks found by our peak discovery pipeline. Note that none of these RBPs had previously known motifs in the two most relevant motif databases: RBPDB [6] and ATtRACT [53]; and only one RBP (EIF4G) has a motif described in the two recent publications on ENCODE eCLIP [54] and RBNS [55].

**Table 3:** ENCODE RBPs analyzed by SARNAclust.

RBP	Domains	#Peaks
AGGF1	G-patch	2207
AKAP8L	C2H2 (2)	2307
DGCR8	DRBM (2)	1929
DKC1	PUA	1573
DROSHA	DRBM	1473
EFTUD2	Tr-type G	2557
EIF3D	RNA-gate	12159
EIF4G2	MIF4G	6415
FAM120A	RNA-binding	27826
FASTKD2	RAP	380
ILF3	DRBM (2), DZF	3410
NKRF	R3H	10681
SMNDC1	Tudor	258
TBRG4	RAP	196

We found motifs for several proteins, with results dependent on the choice of options (see S2 Table). However, analysis of the data under the GraphProt algorithm (equivalent to SARNAclust with option 2) was unable to find any clusters for all but 2 RBPs. Similarly, RNAcontext yielded motif predictions with low Area Under the Receiver Operating Curve values, ranging from 0.111 (NKRF) to 0.546 (AKAP8L), and most motifs had low sequence complexity and information content. In contrast, SARNAclust with options 9, 10 and 11 was able to find clusters almost for all RBPs (all of them for option 9, all but 1 for option 11 and all but 5 for option 10). We focused on clusters with sequence conservation signal in addition to structural information, as those without sequence information would be more difficult to interpret and experimentally validate. This is also why we removed helicases, as we would expect those to lack sequence conservation. Nevertheless, we were able to find several interesting cases, as described below.

SARNAclust results were consistent with k-mer analysis but provided additional structural context. K-mer overrepresentation results [3] for k = 4,5,6,7,8,9 on these RBPs are shown in S3 Table. Most of the k-mers found were either GU repeats or a stretch of guanines GGGG. SARNAclust was able to find these GU repeat motifs as well simultaneously with structure. However, SARNAclust did not reveal other candidate motifs for those proteins, suggesting that those RBPs with repetitive motifs bind to double stranded structures indiscriminately.

#### Single stranded motifs

Although our main interest was to find motifs with secondary structure, we did find several single-stranded motifs. For instance, both AGGF1 and AKAP8L present a motif consisting of a series of CA repeats that are single-stranded (found with both options 9 and 10). Moreover, for AGGF1 we found a second motif consisting of a CCAU repeat that was also single-stranded. Another set of proteins present motifs containing single-stranded GU repeats. These are DKC1 (for which we also find a cluster where the GU repeat is double-stranded), EFTUD2 and EIF4G. For EIF4G, this GU repeat motif is similar to that reported in both ENCODE analysis of eCLIP [54] and RBNS [55]. However, we also found another motif with greater complexity for EIF4G, namely a single-stranded bipartite motif: GUGUGU-GAGAGA.

#### A novel ILF3 motif

**Fig 6:**
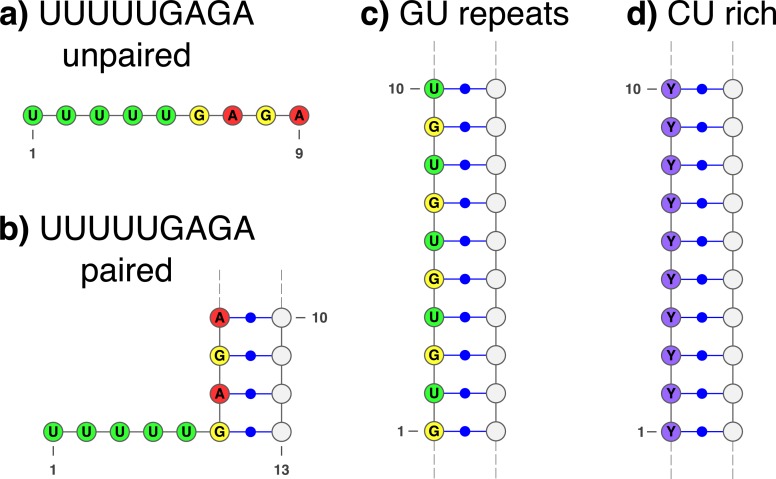
SARNAclust motifs for ILF3. SARNAclust finds 4 different clusters: a) UUUUUGAGA single-stranded motif; b) UUUUUGAGA where GAGA belongs to a stem region; c) GU repeats and d) CU rich region.

Most interestingly we found several motifs for ILF3, including one with both secondary structure and sequence information. These motifs are shown in Fig 6. Note that the motif in Fig 6A is very similar to the one found for the splicing regulator HNRNPC (See Fig 2F in [56]). Therefore, we downloaded the HNRNPC eCLIP data from ENCODE and ran it through our peak discovery pipeline and applied SARNAclust. Indeed, we found the same motif as the one in Fig 6A. We did not find the slightly different motif depicted in Fig 6B, indicating that the 6a version is the common motif.

### Validation of a novel motif for ILF3

Because of the strong predictive motif for ILF3 from SARNAclust, as evidenced by its clusters and signal for double-strandedness, we next used the RBNS approach to validate these motifs. ILF3 is known to be involved in many processes such as transcription, translation, regulation of cell cycle or viral replication [57]. However splicing has only recently been reported as a possible function [58], and confirmation of this HNRNPC-like motif would shed light on a potentially novel function. We therefore used RBNS to test the binding of the predicted motifs with ILF3 and whether it requires a specific RNA structure. Using RNAiFold we designed sequences for 4 different perturbations of the motif as shown in S4 Table (19, 2680, 2680, and 2680 sequences of each motif class respectively). For each motif class, we attempted to generate a few thousand sequences. However, only 19 designed sequences were obtained for the UUUUUGAGA-unpaired motif class due to the fact that unpaired structures tend to have higher free energies than paired structures (see Methods). Similarly as for SLBP, we performed RBNS with purified GST-SBP-ILF3 using an RNA pool based on the motifs in S4 Table.

**Fig 7:**
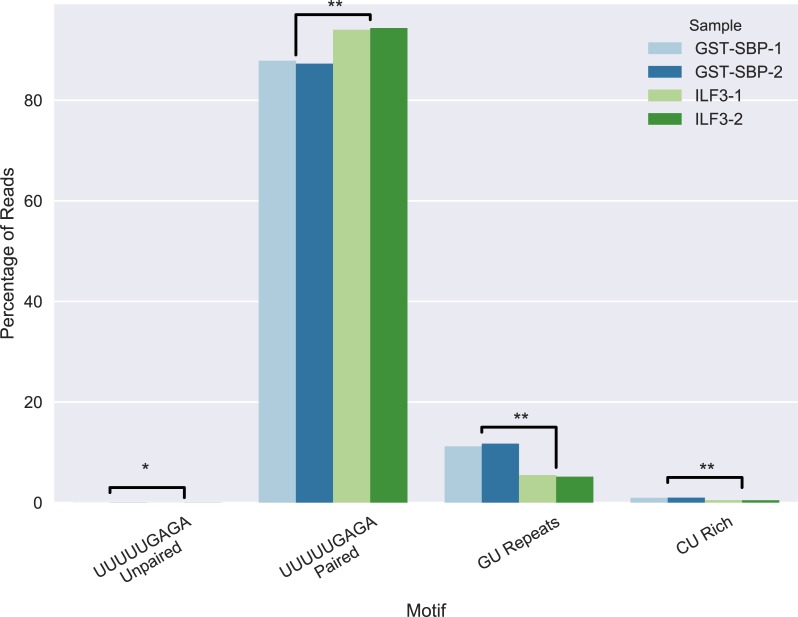
RBNS-like for ILF3 motifs. Percentage shift in the sequences of each cluster of RNAs for ILF3 RNA-bind-n-seq. GST-SBP samples are used as a non-specific binding control. * indicates p < 0.05, ** indicates p < 0.005 assessed by T-test.

Fig 7 shows the shift in percentage of reads of each type and its difference between GST-SBP-1, GST-SBP-2 non-specific binding controls and ILF3-1, ILF3-2 samples. Only the motif UUUUUGAGA-paired exhibited a significant positive shift from the control (p-val<0.005 using the t-test), supporting the novel motif. We also used DEseq to analyze differential representation of sequences in the ILF3 bound and unbound pools (S4 Data). Only sequences from the UUUUUGAGA-paired motif showed significant binding to ILF3, confirming and specifying the computationally discovered motif. 1551 out of 2680 sequences showed increased binding (T-test, p-adj<0.05), while only 6 sequences showed decreased binding. These targeted RBNS results suggest that among the motifs tested, that the UUUUUGAGA-paired shows the strongest binding. In comparison, we observed no significant enrichment or depletion in binding from any of the 19 sequences representing the UUUUUGAGA-unpaired motif. The paired and unpaired sets of sequences each spanned a range of base compositions. The only systematic difference in the two sets was that all of the unpaired motif sequences contained a poly-A sequence that bound to the UUUUU region while preventing pairing of the GAGA region. This is likely a structural constraint, though we cannot rule out that the polyA sequence could also have a sequence-dependent effect on binding. Because the sequences from all motif groups were incubated with the protein at the same time, we note that the lack of enrichment of other motifs is a comparative effect impacted by the stronger binding of UUUUUGAGA-paired motif sequences. Another factor is that RNA Bind-n-Seq does not capture indirect binding interactions mediated through multiprotein complexes, which may be relevant for some of the other motifs. A special case is the GU repeat motif predicted by SARNAclust for ILF3 (GU-repeats motif in S2 Table), which did not show enriched binding. GU-rich motifs were predicted for many other ENCODE RBPs as well ([54]and S3 Table), and we speculate that the presence of such sequences in CLIP data may be due to experimental noise.

These results indicate that ILF3 binds to a UUUUUGAGA motif with most nucleotides in double-stranded regions. They also suggest a relationship between ILF3 and HNRNPC, which has been reported to have a similar motif, though that motif was reported to be single stranded. To explore this further, we analyzed the overlap of ILF3 and HNRNPC peaks in ENCODE eCLIP data. Overall, ILF3 has 822 peaks that fall in anti-sense Alus. 322 of these peaks are shared with HNRNPC peaks, and 146 of those (45%) contain the UUUUUGAGA sequence. Conversely, ILF3 has 285 peaks in anti-sense Alus covering a UUUUUGAGA sequence, and 146 of such peaks (51%) are shared with HNRNPC. Thus from both perspectives a substantial fraction of sites overlap between ILF3 peaks, HNRNPC peaks, and UUUUUGAGA sequences within anti-sense Alus. Our motif analysis predicts 67 of the 146 common sites (46%) to have the paired version of the UUUUUGAGA motif, suggesting there may be some flexibility in RNA structure at the overlapping sites.

## Discussion

SARNAclust is a novel computational method that can effectively process and analyze data from CLIP experiments in order to predict RNA motifs likely to bind individual proteins. A key novelty of SARNAclust is that it can assess RNA binding motifs at the level of the complete RNA structure, rather than only taking into account abstractions of structural context. The SARNAclust approach of clustering rather than classifying distinguishes it from prior methods, allowing it to identify motifs even without training data. This is an important aspect for CLIP-seq, for which the specificity of experimental measurements is not well understood due to diverse effects such as multiple binding modalities and sources of noise.

Application of SARNAclust and the new RBNS validation approach allowed us to experimentally verify ILF3 binding to a newly predicted UUUUUGAGA motif. The signal for this was distinct from repetitive GU or CU motifs, supporting the idea that those repetitive sequences are not true binding sequences. More broadly, SARNAclust allowed us to investigate the relative importance of structure, which has been challenging for RNA-protein interactions, and we found that structure significantly affected the RBNS results for both SLBP and ILF3. Structural changes to each of several components of the SLBP motif reduced binding, and the new motif for ILF3 exhibited a bias for double-strandedness.

Although identification of RBPs that bind to multiple motifs will require further investigation, the multi-domain structure of many RBPs suggest this is a likely possibility. The combination of SARNAclust and our target RBNS validation already allows us to separate multiple distinct signals from noise, making it suited to this ongoing challenge. In contrast, other methods have more difficulty in resolving multiple signals simultaneously. In addition to our results on synthetic motifs, we found that when we selected 1000 ILF3 peaks at random and inputted them to RNAcontext, we found no similar motif to the ones output by SARNAclust (S6 Fig).

The similarity of the new ILF3 motif to that for HNRNPC is intriguing, as it was shown in [56] that HNRNPC competes with another protein U2AF2 for binding of 3’ splice sites to regulate the inclusion/exclusion of exons. They concluded that HNRNPC prevents inclusion of cryptic exons while U2AF2 promotes it, with RBP binding often occurring in antisense Alu elements. Based on this competition, we would expect U2AF2 to have a similar binding site to HNRNPC. However, the predicted motif for HNRNPC is much more similar to that for ILF3 than it is to the predicted *U2AF2* motif (Table 3). We speculate that ILF3 might compete with either HNRNPC or U2AF2 for binding of similar regions.

In this paper we have introduced a new pipeline with a powerful clustering algorithm SARNAclust for analyzing CLIP data in order to cluster CLIP peaks into different binding motifs. We have verified the effectiveness of SARNAclust on synthetic data and used RNA Bind-n-Seq to experimentally validate predictions for new and known motif predictions from ENCODE data. These studies included surveying over different biophysical models and clustering thresholds to identify those likely to work best for real datasets (i.e. options 9,10, 11 at clustering thresholds 0.3–0.55). We have also shown the utility of our RBNS approach by validating its results using gel shift experiments. Still we are we are cognizant of the fact that different RBPs will vary in binding affinity and modality, particularly those with different types of RNA recognition motifs, and further studies will be needed to confirm the generality of these methods for all RBPs. In the future and as more eCLIP data sets for double-stranded binding RBPs become available, we expect SARNAclust will be a valuable tool to discover new motifs, to probe the combinatorial interactions of RNA-binding proteins, and to elucidate their functional importance.

## Methods

### ENCODE data

ENCODE data (www.encodeproject.org) correspond to a set of CLIP experiments described as enhanced CLIP (eCLIP), which modifies the iCLIP method to include improvements in library preparation of RNA fragments. See [52] for details. All data were downloaded through the ENCODE Project website. For proteins with more than one experimental cell type, we used the data from the K562 female cell line.

### Computational pipeline

#### Bam to peaks file processing

We calculated the set of clusters or peaks for each RBP by running pyicoclip on the ENCODE bam files (2 replicates each). The software pyicoclip is part of the pyicoteo software for analysis of high-throughput sequencing data [59] (available at https://bitbucket.org/regulatorygenomicsupf/pyicoteo). Pyicoclip implements the modified False Discovery Rate approach proposed in [3] to determine significant clusters in a list of genomic regions. Pyicoclip implementation, together with the pyicoteo software, offers a flexible and effective framework for the processing and analysis of different types of CLIP-Seq data, with or without associated controls. We chose pyicoteo for its speed and because its modular architecture allowed us to adapt the CLIP-Seq analyses for data standardization. In order to generate a final set of peaks for each RBP, we used peaks that overlapped both replicates and subtracted peaks overlapping with the control. We chose this approach rather than using enrichment thresholds in order to minimize noise from any systematic measurement biases. For each peak we tracked the gene it overlapped, the type of region within the gene, and the genomic sequence.

#### Structure prediction

All structures are predicted using RNAfold from the Vienna Package on the exact binding region without extending it. We chose this after considering an alternative approach with more flexibility in handling edge effects. The other approach would calculate base pairing probabilities of the binding region with 100nt (an adjustable parameter) extended on each end, followed by a form of Nussinov folding. However, we found that such an approach led to comparable results as when RNAfold was simply applied to the binding region alone.

#### Clustering algorithm

The clustering algorithm is the main component in our pipeline. Given a set of RNA sequences along with their predicted secondary structures, it identifies clusters of similar RNAs by encoding both sequence and structure as a graph, and using the EdEN kernel similarly as in GraphClust. The pipeline accepts several parameters to control both the graph transformation and the clustering. It also allows for the use of only sequence information in a sliding window fashion. The clustering algorithms supported are: K-means, Mean Shift, DB-Scan, Affinity Propagation and Spectral Clustering from sklearn package (http://scikit-learn.org/stable) and Density Clustering [60] in an in-house implementation. Details are in the full implementation available at the Github site. For the clustering of the synthetic motif data we used the EdEN graph kernel with DB-SCAN, surveying over possible values for the DB-Scan parameter *threshold* (http://scikit-learn.org/stable/modules/generated/sklearn.cluster.DBSCAN.html), which specifies the minimal similarity for two data points to be in the same cluster. Other parameter choices were *radius* = 2 and *distance* = 2 with *min_samples* = 10 (see [38]).

For the ENCODE predictions, to balance memory constraints we limited peaks to those with length between 10 and 80 nucleotides, and we broke down separate stem loops into different derived peaks. Moreover, only 1400 sequences could only be considered at each time, so we extended SARNAclust to deal with multiple iterations and merge similar clusters at the end.

#### SARNAclust runtime

In our benchmarking, using DBSCAN for all RBPs with more than 1400 peaks, SARNAclust takes an average of 6 minutes per iteration. Usage times will vary somewhat for different systems, since SARNAclust uses disparate external tools, such as EdEN for the graph transformation, sklearn for the clustering and locarna for the alignment of the clusters. Inside SARNAclust we limit the number of peaks to be analyzed per iteration (1400) due to memory constraints. Therefore, only RBPs with very few peaks end up taking less time per iteration. Runtimes are also impacted by the length of the peaks, the complexity of the predicted structure, the choice of transformation and EdEN parameters, and the choice of clustering algorithm.

#### Clustering quality measures

The Adjusted Rand Index (ARI) (http://scikit-learn.org/stable/modules/clustering.html#clustering-performance-evaluation) measures the similarity of the two assignments ignoring permutations versus random expectations; Adjusted Mutual Information (AMI) measures the agreement of the two assignments normalized against random expectations; The Homogeneity Score (HS) quantifies the fact that each cluster should contain only members of a single class, while the Completeness Score (CS) is based on whether all members of a given class are assigned to the same cluster; V-measure score (VMS) is the harmonic mean between HS and CS. The Fowlkes-Mallows score (FMS) computes the accuracy of overlap between the found clusters and the original benchmarks, with higher FM values indicating greater overlap.

#### RNAiFold to generate candidates

In order to generate candidate RNA sequences for the RBNS experimental validation we used the RNA inverse folding software RNAiFold [43]. Given a sequence/structure RNA motif, we attempted to generate thousands of sequences that fold into the given secondary structure and maintain the given sequence constraints. Sequences generated by RNAiFold were used in the design of the RBNS pool. Moreover, we used RNAiFold to generate sequences corresponding to perturbations of the SLBP and ILF3 motifs. This was done by altering constraints and re-running RNAiFold, e.g. for SLBP we moved a sequence motif from a stem loop to a bulge to generate a pool of sequences that would test whether location of the sequence motif within the structure affected binding. One constraint to this design process was that not all motifs were equally easy to design given their sequence/structure requirements and the need to use specific primers. Sequences for the ILF3 UUUUUGAGA-unpaired motif were particularly difficult to design because structures with base pairing tend to have lower energy than unpaired structures, so we were only able to design 19 sequences for that motif.

### Experimental pipeline

#### In vitro protein expression and purification

A previously generated pGEX6P1-based expression vector containing streptavidin binding peptide (SBP)-tagged ILF3 was used for ILF3 binding experiments. For SLBP experiments, the protein coding sequence for SLBP was codon optimized for *E*. *coli* expression using the IDT Codon Optimization Tool and Gibson assembled into pGEX6P1-SBP, which enhanced solubility compared to the human sequence. Each plasmid was transformed into Rosetta(DE3) pLysS *E*. *coli*. Protein expression was induced with 1mM isopropylthiogalactoside (IPTG) and grown for 4 hours at 16°C. Soluble protein was extracted from the bacteria using the Qproteome Bacterial Protein Prep Kit (QIAGEN). The proteins were then affinity purified using Glutathione Sepharose 4B and eluted in a buffer containing 0.2% Triton X-100 and concentrated using Corning Spin-X UF with a 10 kDa molecular weight cutoff (MWCO). Proteins were then equilibrated into RBNS binding buffer (25mM Tris pH 7.5, 150mM KCl, 0.1% Tween, 0.5 mg/mL BSA, 3mM MgCl_2_, 1mM DTT) using Zeba desalting columns 7KDa MWCO. Purified proteins were then frozen at -80°C for short-term storage. Protein concentrations were obtained using Pierce BCA Protein Assay Kit. Protein purity was assessed using SDS-PAGE.

#### RNA pool generation for RNA Bind-N-Seq

Oligonucleotide sequences were ordered from CustomArray Inc. in a 12,472 oligo pool. PCR was used to amplify the ILF3 pool (5’-CCCATAATACTTGTCCCG*-*3’ and 5’-TAATACGACTCACTATAGGG-3’) and the SLBP pool (5’-CTTGACTGCGAGCTGTTGA-3’ and 5’-TAATACGACTCACTATAGGTCACGTC-3’). *In vitro* transcription of the oligo pool was performed using an AmpliScribe T7 High Yield Transcription Kit. The RNA was purified by lithium chloride precipitation and resuspended in RBNS binding buffer.

#### RNA Bind-N-Seq

RBNS was performed as described in [49]. 27 nM of each protein was incubated with 750 pM of RNA. RNA was reverse transcribed using SuperScript III Reverse Transcriptase and a primer containing a 10 nucleotide barcode for SLBP (5’- GTGACTGGAGTTCAGACGTGTGCTCTTCCGATCTNNNNNNNNNNCTTGACTGCGTGCTGTTGA -3’) and ILF3 (5’-GTGACTGGAGTTCAGACGTGTGCTCTTCCGATCTNNNNNNNNNNCCCATAATACTTGTCCCG-3’). PCR was performed to amplify cDNA derived from the RBNS RNAs and attach Illumina flow cell binding sequences and indices (5’- AATGATACGGCGACCACCGAGATCTACAC-i5_index-ACACTCTTTCCCTACACGACGCTCTTCCGATCT-3’ and 5’- CAAGCAGAAGACGGCATACGAGAT-i7_index-GTGACTGGAGTTCAGACGTGTGCTCTTCCGATCT-3’). DNA was sequenced on a MiSeq using a 200 cycle paired end kit.

#### RBNS data analysis

FLASH was used to join paired end reads that intersected, which is expected for each of the sequences tested [61]. Reads that contained the anticipated primer sequences were aligned using HISAT2 [62]. Reads aligning to the same sequence and containing the same 10 nucleotide random sequence were collapsed into one read using a custom python script. The resulting counts were input to DEseq for analysis [51].

### Gel shift experiments

Probes were *in vitro* transcribed and biotinylated using the Pierce RNA 3’ end biotinylation kit. 1 nM of biotinylated probe was incubated with or without 320 nM GST-SBP-SLBP in binding buffer consisting of 10 mM HEPES (pH 7.3), 20 mM KCl, 1 mM MgCl_2_, 20 mM DTT, 5% glycerol. The incubation period was 30 minutes, followed by gel electrophoresis on a native TBE 4% polyacrylamide gel and transfer to a nylon membrane, all at 4°C.

Membranes were processed using the ThermoFisher Scientific Chemiluminescent Nucleic Acid Detection Module Kit. Images were captured on a Kodak ImageStation 4000MM Pro.

## Supporting information

S1 Fig Pipeline for CLIP peak detection. Given bam files for samples and control, pyicoclip is used to detect significant peaks in each file. Afterwards, we filter those peaks that do not appear in all the samples and remove those that can be found in the control. The resulting peaks are annotated and the sequences for them (+/- 100 flanking nucleotides) are retrieved.
(DOCX)

S2 Fig Graphical representation of all the graph transformation options. SF2.k corresponds to graph transformation option k as explained in the main text. Examples are shown for sequence/structure:
GGGGAAACCAACCUGU
((((. . .))..)) . . .
(DOCX)

S3 Fig Sequence logo and structural context found by RNAcontext on the synthetic dataset. (DOCX)

S4 Fig SDS-PAGE gel showing the result of GST affinity purification of GST-SBP, GST-SBP-SLBP and GST-SBP-ILF3. Proteins of this purity were used in RBNS and gel shift assays.
(DOCX)

S5 Fig Logo for SLBP consensus motif sequences that a) show enriched binding and b) do not show enriched binding.
(DOCX)

S6 Fig Sequence logo and structural context found by RNAcontext on ILF3. (DOCX)

S1 Table Four different classes of designed sequences used for the RNA Bind-N-Seq validations for SLBP. (DOCX)

S2 Table Comparison of RBP motifs found per graph transformation option and DBSCAN threshold. (DOCX)

S3 Table Best k-mer found for all RBPs considered. For each RBP, the z-score is given for the k-mer with the maximal value of Z-score*k.
(DOCX)

S4 Table Four different classes of designed sequences used for the RNA Bind-N-Seq validations for ILF3. (DOCX)

S1 Data All motif and random sequences generated for benchmarking SARNAclust. (XLSX)

S2 Data Clustering quality measures for each (Adjusted Rand Index (ARI), Adjusted Mutual Information (AMI), Homogeneity Score (HS), Completeness Score (CS), V-measure score (VMS) and Fowlkes-Mallows score (FMS)) for all thresholds and options as described in the benchmarking section. (XLSX)

S3 Data DEseq2 results for all sequences generated to test SLBP candidate motifs. (XLSX)

S4 Data DEseq2 results for all sequences generated to test ILF3 candidate motifs. (XLSX)
