# Supplementary material for: SARNAclust: Semi-automatic detection of RNA protein binding motifs from immunoprecipitation data
Source: PLoS Comput Biol. 2018 Mar 29;14(3):e1006078. doi: 10.1371/journal.pcbi.1006078 (PMC5892938; doi:10.1371/journal.pcbi.1006078)
Supplement: S1 Fig — Given bam files for samples and control, pyicoclip is used to detect significant peaks in each file. Afterwards, we filter those peaks that do not appear in all the samples and remove those that can be found in the control. The resulting peaks are annotated and the sequences for them (+/- 100 flanking nucleotides) are retrieved. (DOCX) [file pcbi.1006078.s001.docx]

**S1 Fig.**

**
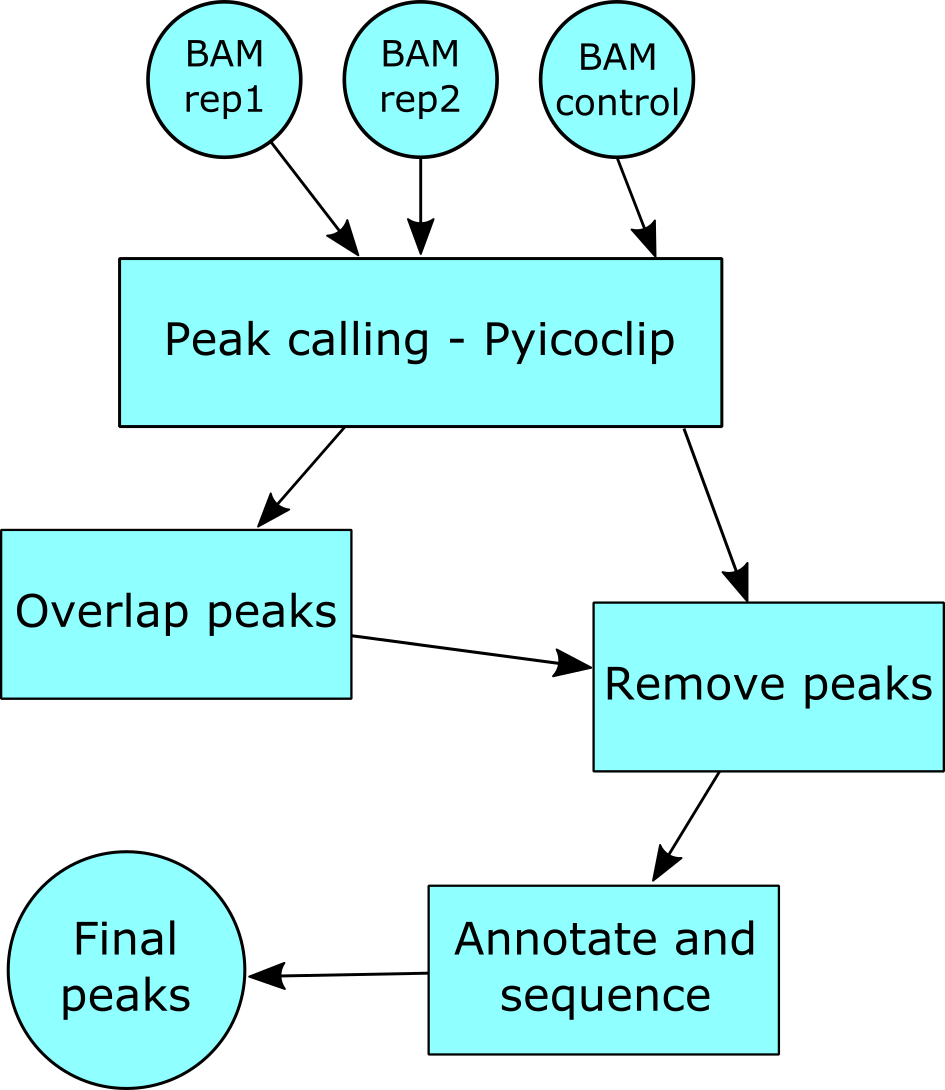
**

Legend: Pipeline for CLIP peak detection. Given bam files for samples and control, pyicoclip is used to detect significant peaks in each file. Afterwards, we filter those peaks that do not appear in all the samples and remove those that can be found in the control. The resulting peaks are annotated and the sequences for them (+/- 100 flanking nucleotides) are retrieved.
