# Supplementary material for: SARNAclust: Semi-automatic detection of RNA protein binding motifs from immunoprecipitation data
Source: PLoS Comput Biol. 2018 Mar 29;14(3):e1006078. doi: 10.1371/journal.pcbi.1006078 (PMC5892938; doi:10.1371/journal.pcbi.1006078)
Supplement: S2 Fig — SF2.k corresponds to graph transformation option k as explained in the main text. Examples are shown for sequence/structure: GGGGAAACCAACCUGU ((((. . .))..)) . . . (DOCX) [file pcbi.1006078.s002.docx]

**S2 Fig.**

**1**

**
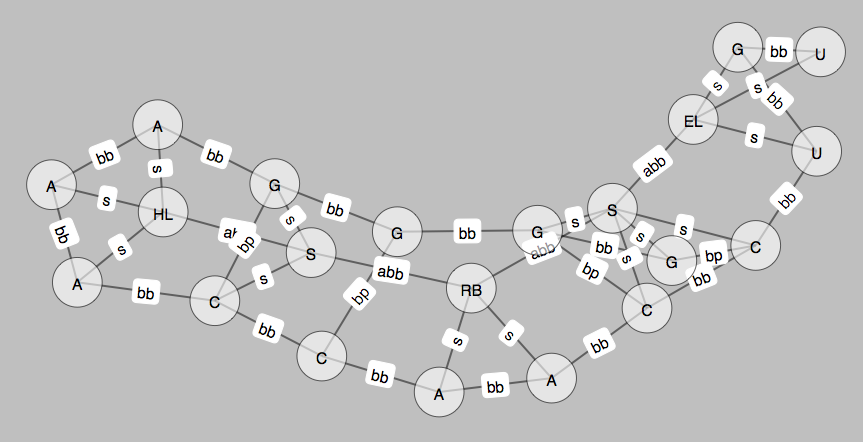
**

**2**

**
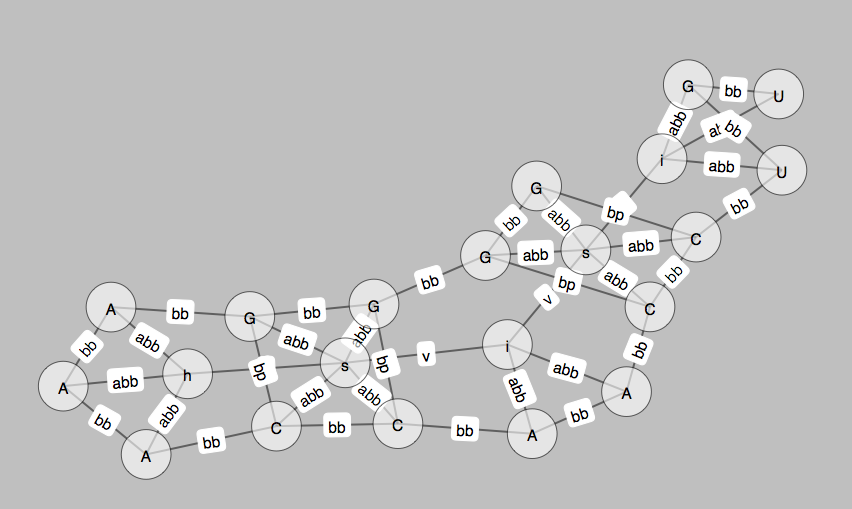
**

**3**

**
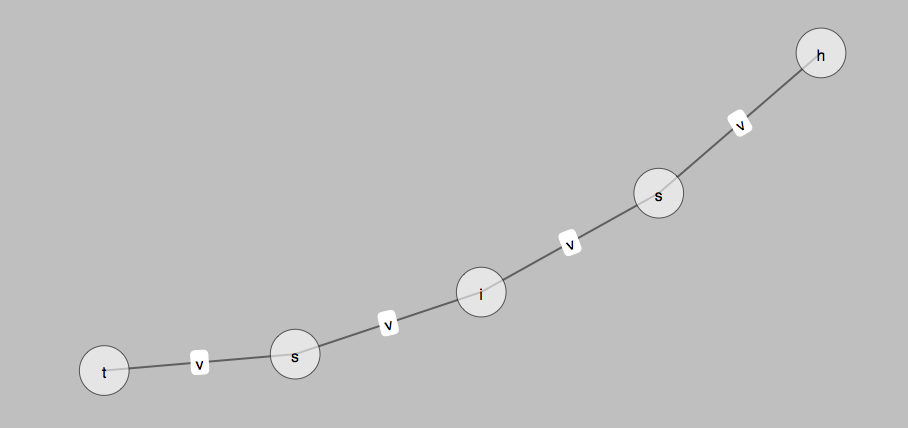
**

**4**

**
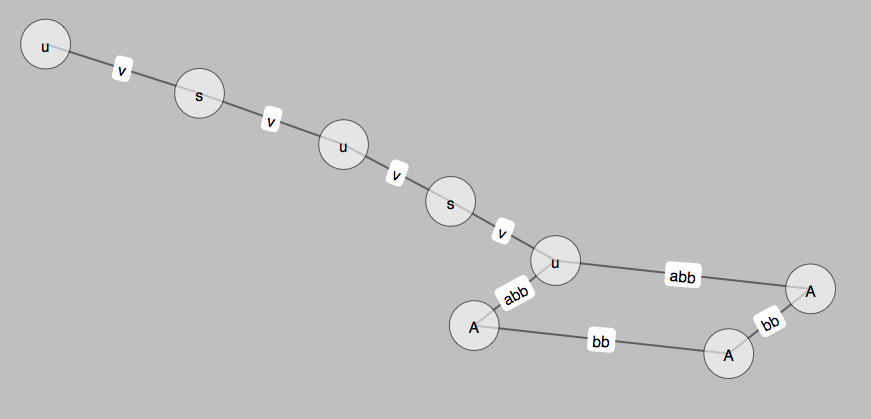
**

**5**

**
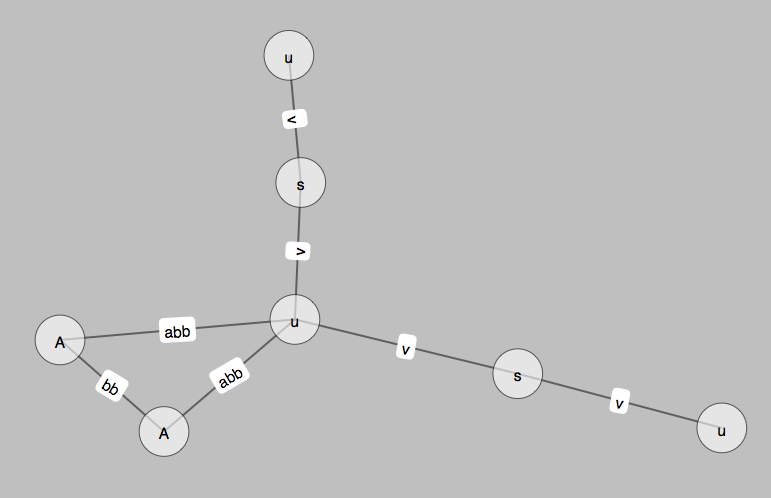
**

**6**

**
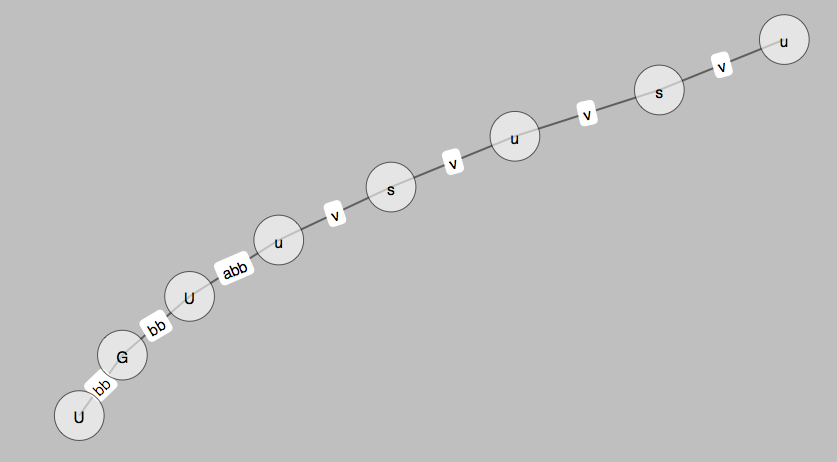
**

**7**

**
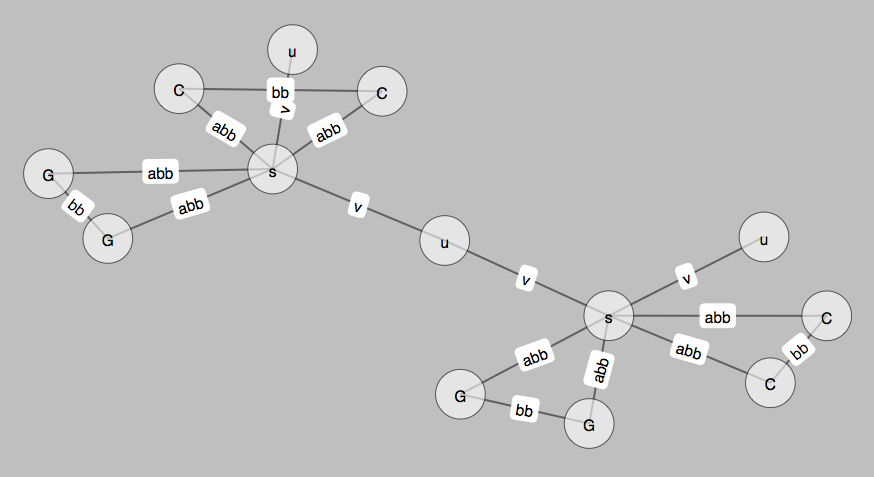
**

**8**

**
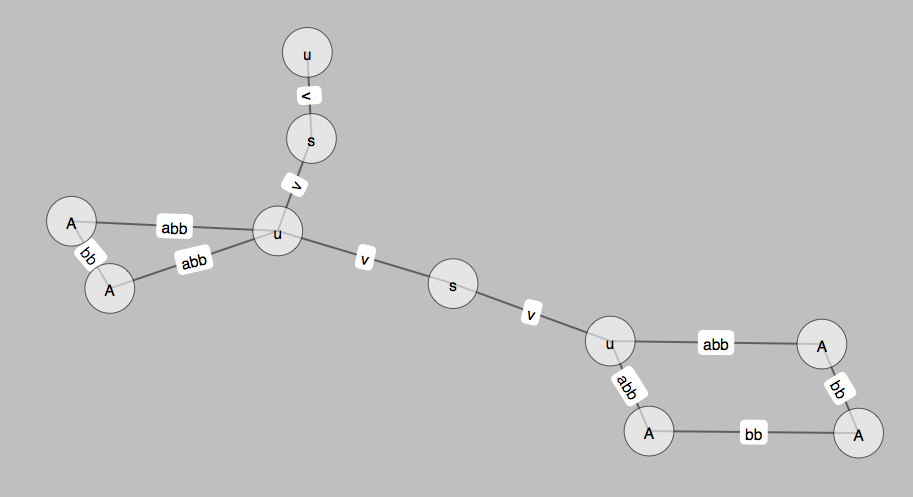
**

**9**

**
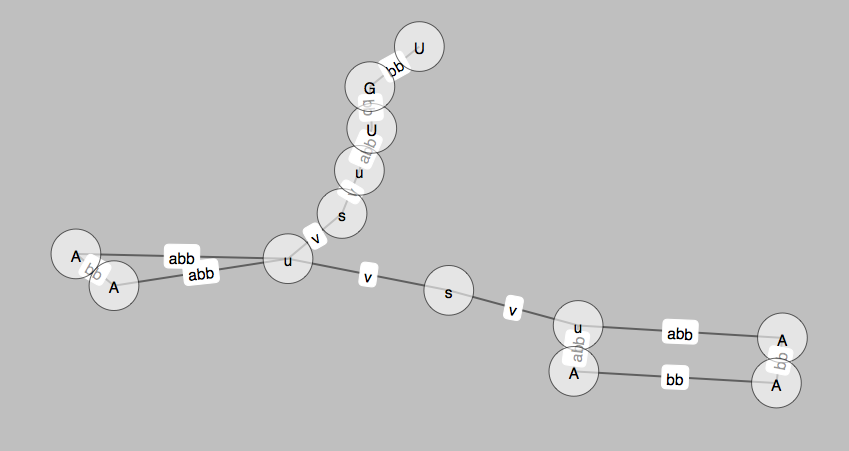
**

**10**

**
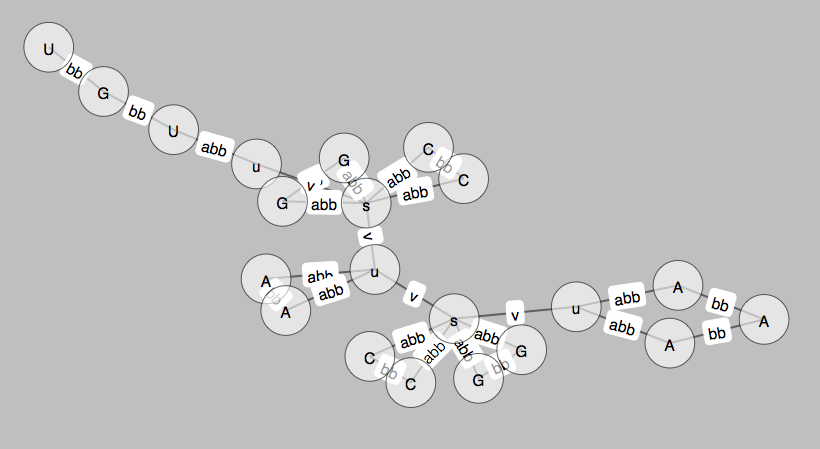
**

**11**

**
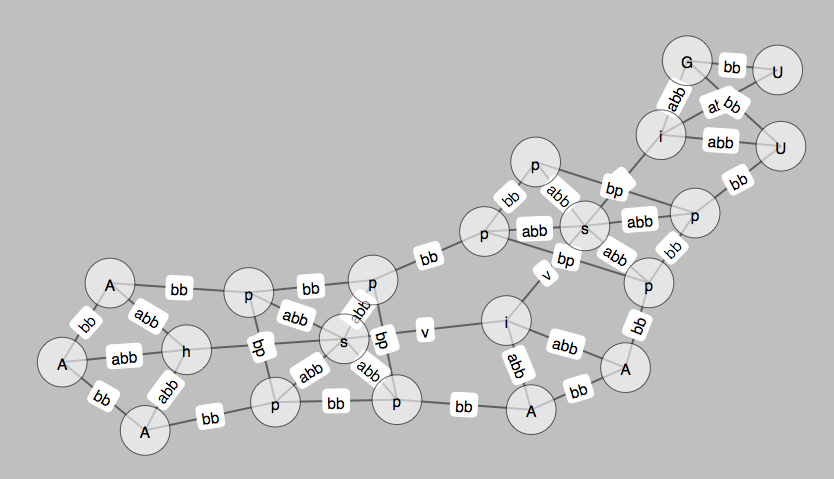
**

Legend: Graphical representation of all the graph transformation options. SF2.k corresponds to graph transformation option k as explained in the main text. Examples are shown for sequence/structure

GGGGAAACCAACCUGU

((((...))..))...
