# Supplementary material for: SARNAclust: Semi-automatic detection of RNA protein binding motifs from immunoprecipitation data
Source: PLoS Comput Biol. 2018 Mar 29;14(3):e1006078. doi: 10.1371/journal.pcbi.1006078 (PMC5892938; doi:10.1371/journal.pcbi.1006078)
Supplement: S3 Fig — (DOCX) [file pcbi.1006078.s003.docx]

**S3 Fig**

**A**

**
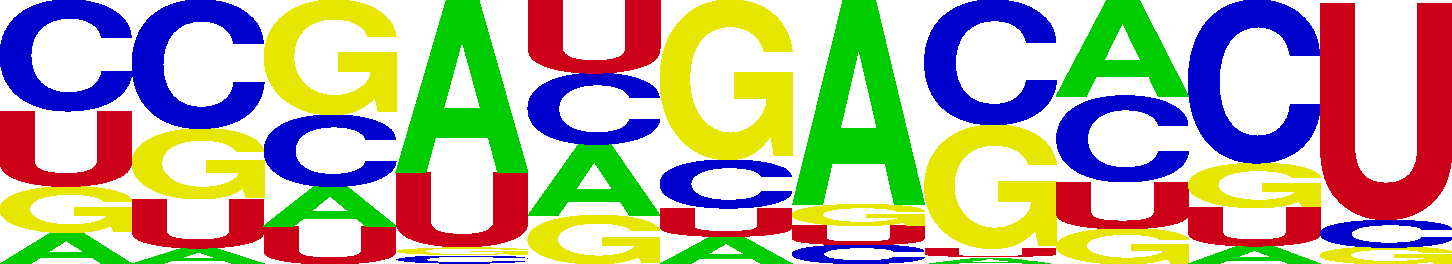
**

**B**

**
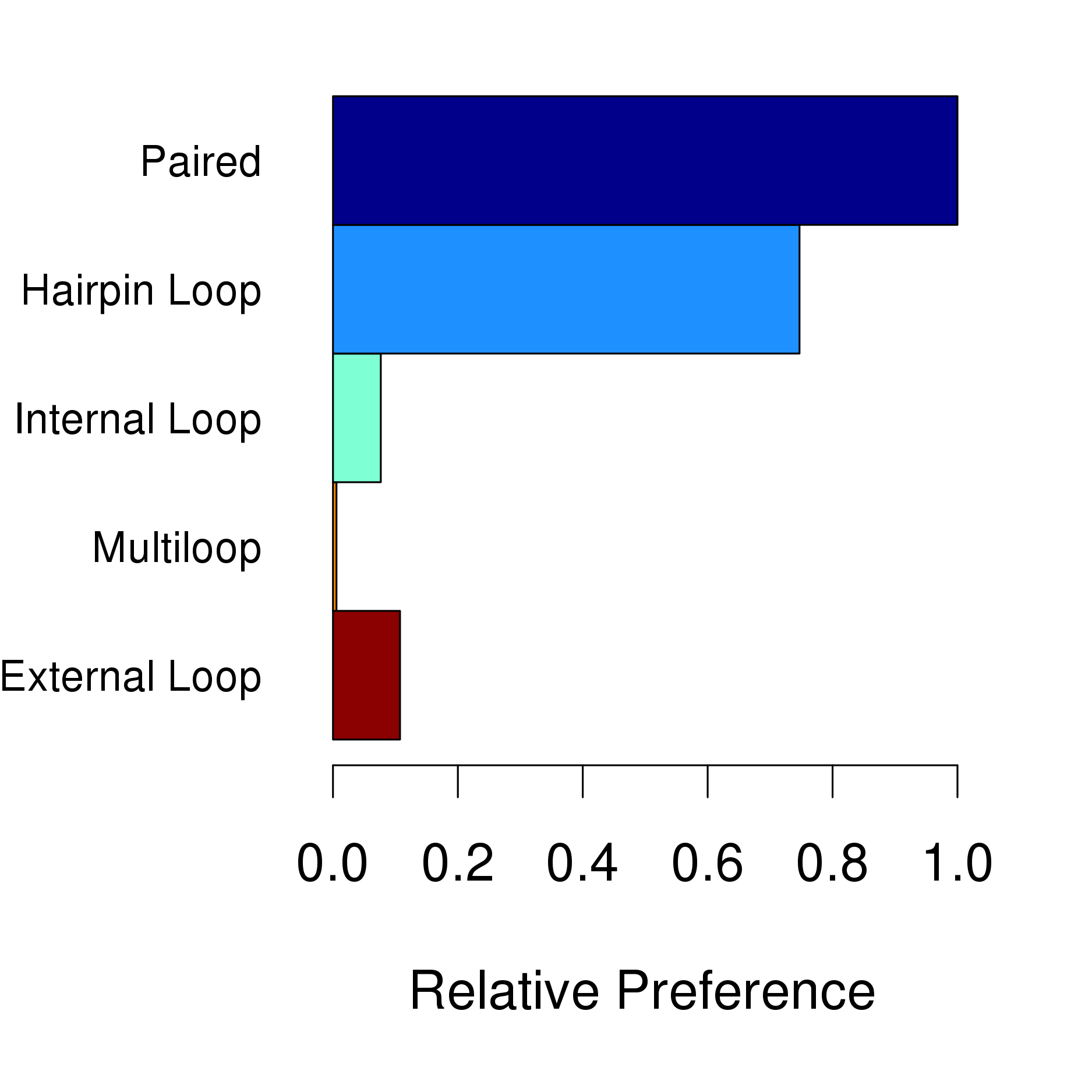
**

Legend: (A) Sequence logo and (B) structural context found by RNAcontext on the synthetic dataset.
