# Supplementary material for: SARNAclust: Semi-automatic detection of RNA protein binding motifs from immunoprecipitation data
Source: PLoS Comput Biol. 2018 Mar 29;14(3):e1006078. doi: 10.1371/journal.pcbi.1006078 (PMC5892938; doi:10.1371/journal.pcbi.1006078)
Supplement: S4 Fig — Proteins of this purity were used in RBNS and gel shift assays. (DOCX) [file pcbi.1006078.s004.docx]

**S4 Fig**

**
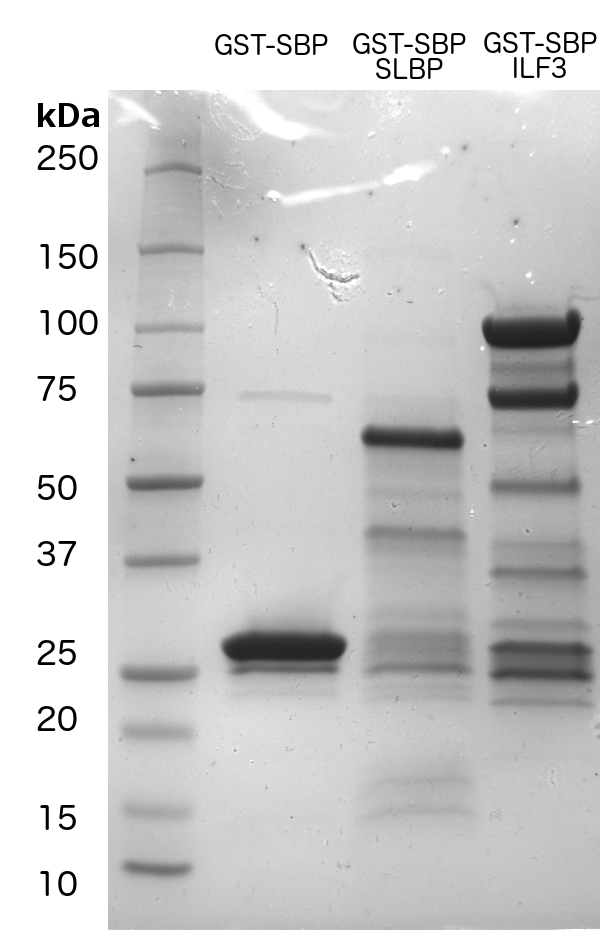
**

Legend: SDS-PAGE gel showing the result of GST affinity purification of GST-SBP, GST-SBP-SLBP and GST-SBP-ILF3. Proteins of this purity were used in RBNS and gel shift assays.
