# Supplementary material for: SARNAclust: Semi-automatic detection of RNA protein binding motifs from immunoprecipitation data
Source: PLoS Comput Biol. 2018 Mar 29;14(3):e1006078. doi: 10.1371/journal.pcbi.1006078 (PMC5892938; doi:10.1371/journal.pcbi.1006078)
Supplement: S5 Fig — Logo for SLBP consensus motif sequences that a) show enriched binding and b) do not show enriched binding. (DOCX) [file pcbi.1006078.s005.docx]

**S5 Fig**

**A**


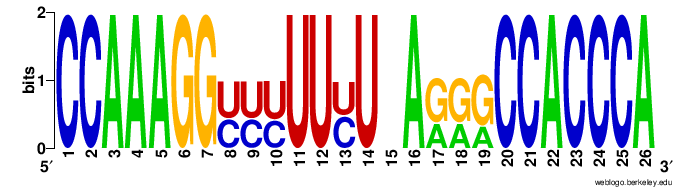


**B**


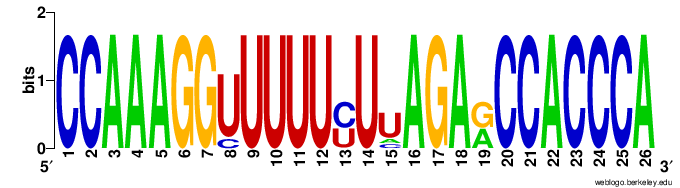


Legend: Logo for SLBP consensus motif sequences that (A) show enriched binding and (B) do not show enriched binding.
