# Supplementary figures and images for: SARNAclust: Semi-automatic detection of RNA protein binding motifs from immunoprecipitation data
Source: PLoS Comput Biol. 2018 Mar 29;14(3):e1006078. doi: 10.1371/journal.pcbi.1006078 (PMC5892938; doi:10.1371/journal.pcbi.1006078)

**S6 Fig**

**A**


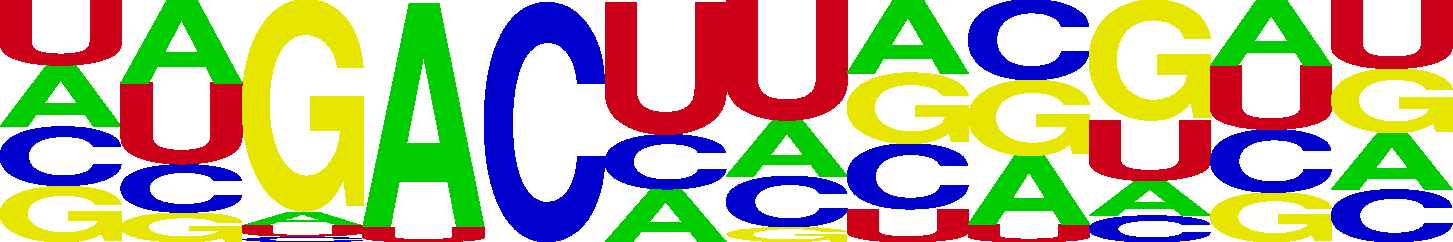


**B**


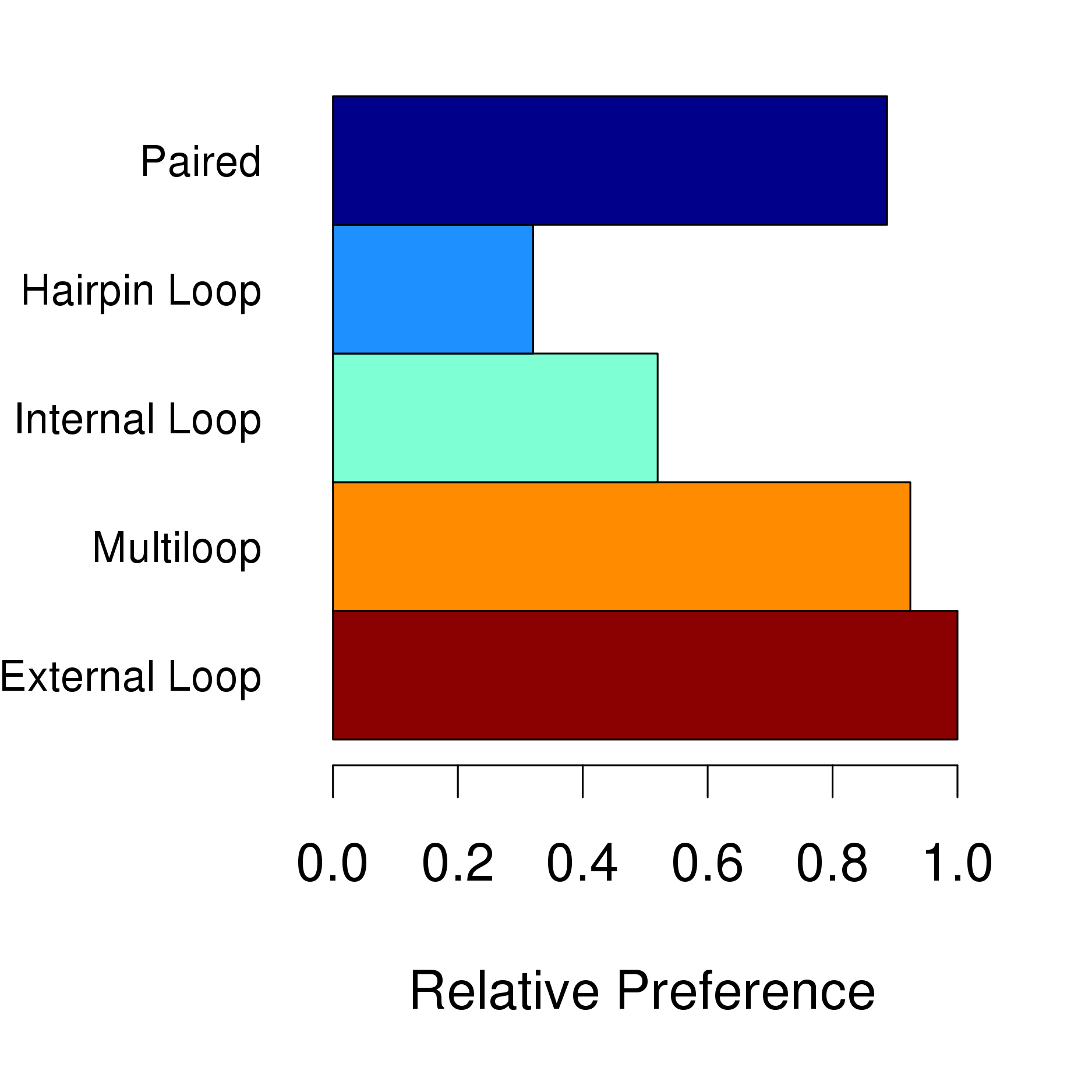


Legend: (A) Sequence logo and (B) structural context found by RNAcontext on ILF3.

Supplement: S6 Fig — (DOCX) [file pcbi.1006078.s006.docx]
