# Supplementary material for: SARNAclust: Semi-automatic detection of RNA protein binding motifs from immunoprecipitation data
Source: PLoS Comput Biol. 2018 Mar 29;14(3):e1006078. doi: 10.1371/journal.pcbi.1006078 (PMC5892938; doi:10.1371/journal.pcbi.1006078)
Supplement: S1 Table — (DOCX) [file pcbi.1006078.s007.docx]

**S1 Table:** Four different classes of designed sequences used for the RNA Bind-N-Seq validations for SLBP.

| **Name** |  |  |
| --- | --- | --- |
| **consensus** | .....((((((....))))))..... | **Structure** |
|  | CCAAAGGYYYUUYUNARRRCCACCCA | **Sequence** |
| **consensusLoopOnly** | .....((((((....))))))..... | **Structure** |
|  | DDBBBHHRRRVUYUNBYYYDDBDDDB | **Sequence** |
| **LooStOnly** | .....((((((....))))))..... | **Structure** |
|  | DDBBBHHRRRVVRVNBYYYDDBDDDB | **Sequence** |
| **loopInBulge** | ....(((((((((....))))....))))).... | **Structure** |
|  | NNNNNNNNNNNNNNNNNNNNNUYUNNNNNNNNNN | **Sequence** |
