# Supplementary material for: SARNAclust: Semi-automatic detection of RNA protein binding motifs from immunoprecipitation data
Source: PLoS Comput Biol. 2018 Mar 29;14(3):e1006078. doi: 10.1371/journal.pcbi.1006078 (PMC5892938; doi:10.1371/journal.pcbi.1006078)
Supplement: S2 Table — (DOCX) [file pcbi.1006078.s008.docx]

**S2 Table:** Comparison of RBP motifs found per graph transformation option and DBSCAN threshold

| **RBP** | **Motif** | **Thresholds** | | | |
| --- | --- | --- | --- | --- | --- |
|  |  | **Option2** | **Option9** | **Option10** | **Option11** |
| AGGF1 | CA* | - | 0.3 | 0.3-0.4 | - |
|  | CCAU* | - | 0.3 | 0.3-0.4 | - |
| AKAP8L | CA* | - | 0.3 | 0.3-0.45 | - |
| DKC1 | ssGU* | 0.5 | 0.4 | 0.4 | 0.4 |
|  | dsGU* | 0.5-0.55 | - | - | 0.4 |
| EFTUD2 | GU* | - | - | - | 0.3 |
| EIF3D | GU* | - | - | - | 0.3 |
| EIF4G | GU* | - | 0.3-0.4 | 0.3-0.4 | 0.3-0.4 |
|  | GUGUGU-GAGAGA | 0.3 | 0.3 | 0.3 | 0.3-0.4 |
| ILF3 | ssUUUUUGAGA | - | 0.3-0.4 | 0.35-0.4 | - |
|  | dsUUUUUGAGA | - | 0.35 | 0.35-0.4 | - |
|  | GU* | - | - | - | 0.4 |
|  | CU-rich | - | 0.45 | 0.35 | - |
