# Supplementary material for: SARNAclust: Semi-automatic detection of RNA protein binding motifs from immunoprecipitation data
Source: PLoS Comput Biol. 2018 Mar 29;14(3):e1006078. doi: 10.1371/journal.pcbi.1006078 (PMC5892938; doi:10.1371/journal.pcbi.1006078)
Supplement: S3 Table — For each RBP, the z-score is given for the k-mer with the maximal value of Z-score*k. (DOCX) [file pcbi.1006078.s009.docx]

**S3 Table:** Best k-mer found for all RBPs considered. For each RBP, the z-score is given for the k-mer with the maximal value of Z-score*k

| **RBP** | **Best k-mer** | **Zscore** |
| --- | --- | --- |
| **AGGF1** | CACACA | 50.76315181 |
| **AKAP8L** | ACACAC | 19.99310723 |
| **DGCR8** | UGUGUGUGU | 3.78345072 |
| **DKC1** | GUGUGUGUG | 54.85391672 |
| **DROSHA** | GGGG | 18.10038977 |
| **EFTUD2** | GUGUGUGUG | 10.01795948 |
| **EIF3D** | GUGUGUGUG | 8.819173439 |
| **EIF4G2** | GUGUGUGUG | 81.26754169 |
| **FAM120A** | GGGG | 121.3133703 |
| **FASTKD2** | GGGG | 16.69354427 |
| **ILF3** | UUUUUGAGA | 8.611818309 |
| **NKRF** | GGGG | 65.97177516 |
| **SMNDC1** | UGUCUG | 3.06627067 |
| **TBRG4** | GUGUGUGUG | 4.918158783 |
