# Supplementary material for: SARNAclust: Semi-automatic detection of RNA protein binding motifs from immunoprecipitation data
Source: PLoS Comput Biol. 2018 Mar 29;14(3):e1006078. doi: 10.1371/journal.pcbi.1006078 (PMC5892938; doi:10.1371/journal.pcbi.1006078)
Supplement: S4 Table — (DOCX) [file pcbi.1006078.s010.docx]

**S4 Table:** Four different classes of designed sequences used for the RNA Bind-N-Seq validations for ILF3. 19, 2680, 2680, and 2680 sequences of each motif type were tested in the experiment, respectively.

| **Name** |  |  |
| --- | --- | --- |
| **uuuugaga Unpaired** | .............................. | **Structure** |
|  | UUUUUUUUUUGAGANNNNNNNNNNNNNNNN | **Sequence** |
| **uuuugaga Paired** | ..........(((((((...)))))))... | **Structure** |
|  | UUUUUUUUUUGAGANNNNNNNNNNNNNNNN | **Sequence** |
| **gu Repeats** | (((((((((((((...))))))))))))) | **Structure** |
|  | KKKKKKKKKKKKKNNNNNNNNNNNNNNNN | **Sequence** |
| **cu Rich** | (((((((((((((...))))))))))))) | **Structure** |
|  | YYYYYYYYYYYYYNNNNNNNNNNNNNNNN | **Sequence** |
